# The COMPASS framework for chronic pain and posttraumatic stress disorder: navigating the transition from threat to safety through yoga

**DOI:** 10.3389/fpubh.2026.1786419

**Published:** 2026-06-24

**Authors:** Belle Zaccari, Steffany Moonaz, Marlysa B. Sullivan

**Affiliations:** 1 Center to Improve Veteran Involvement in Care, VA Portland Health Care System, Portland, OR, United States; 2 Department of Psychiatry, Oregon Health and Science University, Portland, OR, United States; 3 VHA Office of Rural Health, Veterans Rural Health Resource Center, Portland, OR, United States; 4 Southern California University of Health Sciences, Whittier, CA, United States; 5 Atlanta VA Healthcare System, Atlanta, GA, United States; 6 Notre Dame of Maryland University, Baltimore, MD, United States

**Keywords:** chronic pain, framework, posttraumatic stress disorder, salutogenesis, yoga

## Abstract

Chronic pain and PTSD (CP + PTSD) have a high rate of co-prevalence. Existing frameworks and models have driven the development of few simultaneous treatment approaches, though the prevailing psychological models and treatments for this comorbidity are pathogenic, incomplete, and fail to consider the whole-person impact of CP + PTSD. This warrants consideration of novel treatment perspectives and approaches. Salutogenesis offers an alternative paradigm and is a transdiagnostic and transdisciplinary perspective that addresses the complex, multifaceted dimensions of CP + PTSD and supports whole-person well-being. We present the Connectedness, Optimism and hope, Mind–body regulation, Purpose and values, Awareness of the body, Self-efficacy, and Safety (COMPASS) framework to illustrate how a person with CP + PTSD can use yoga practices to navigate experiences perceived as threatening, in order to cultivate safety and resilience. The discussion highlights ways the COMPASS framework can be foundational to interdisciplinary care and utilized by healthcare and research professionals to deliver a whole-person approach to CP + PTSD. We conclude by suggesting next steps for applying COMPASS in clinical and research settings.

## 1 Introduction

Almost a quarter of all adults in the United States (US) live with chronic pain (CP), about a third of whom experience limitations to work and life activities (1). Chronic pain is associated with reduced quality of life, interfering with physical function, work activities, family life, social connection, sleep, and mood, all of which are amplified by the co-occurrence of another mental health disorder (2, 3). Mood disorders like depression and anxiety commonly co-occur with chronic pain, and more than half of US adults with a mood disorder also report CP (4). PTSD alone is associated with global functional impairment and disruptions to quality of life (5, 6). Meta-analytic studies examining pain among individuals with and without PTSD consistently show higher pain intensity, pain catastrophizing, disability, and healthcare utilization when PTSD is present (7). Both chronic pain and PTSD are heterogeneous in their clinical presentations, and additional complexity is present when they co-occur. This entails corresponding clinical implications, challenges, and opportunities for developing intervention strategies and treatment outcomes (8–11).

Among those with chronic pain, the risk and prevalence of posttraumatic stress disorder (PTSD) are elevated due to the bidirectional relationship between the two conditions and shared neurobiology (10, 12). The comorbidity rates of CP + PTSD in the civilian population range from 15 to 35% and rise to 34 to 50% among Veteran populations (7, 13, 14). For those living with CP + PTSD, symptoms are greater than for those with only one or the other condition (15). Evidence-based care suggests an integrated treatment approach that considers the complex interaction between these two conditions (16).

Biological and psychological models of CP + PTSD help explain the development of these conditions and inform treatment. The biopsychosocial model is a well-established framework, introduced as an alternative to the biomedical model in 1977 (17). Cognitive-behavioral models describe the complex relationship between chronic pain and PTSD and highlight catastrophic thinking, avoidance behavior, and pathophysiology (18). Recent research has advanced traditional cognitive-behavioral models by incorporating concepts like biological mechanisms, and values and purpose (19–21). Weaknesses of existing frameworks and models include inconsistent understanding and implementation, reductionism, failure to capture the whole-person implications of CP + PTSD, and therefore, barriers to treatment and recovery (22, 23).

Some weaknesses of existing models have been addressed by mindfulness-based conceptualizations and interventions, like yoga’s *Kosha* model. Yoga practices are mindfulness-based, support a multi-layered understanding of one’s experience that is relevant to CP + PTSD, and increase awareness of one’s internal states (i.e., interoception). Further, yoga’s philosophical components confer additional widespread benefits to whole-person functioning, impacting one’s sense of safety (e.g., feeling resourced enough to encounter stress and perceived threat) and resilience (e.g., being able to adapt after actual or perceived setbacks or stressors). Yoga has been adapted for therapeutic use globally, and a growing body of literature includes studies on the effectiveness of yoga for reducing chronic pain, PTSD, and other clinical psychiatric symptoms (e.g., depression and anxiety), while improving quality of life and whole-person wellness (24–27).

**Figure 1:**
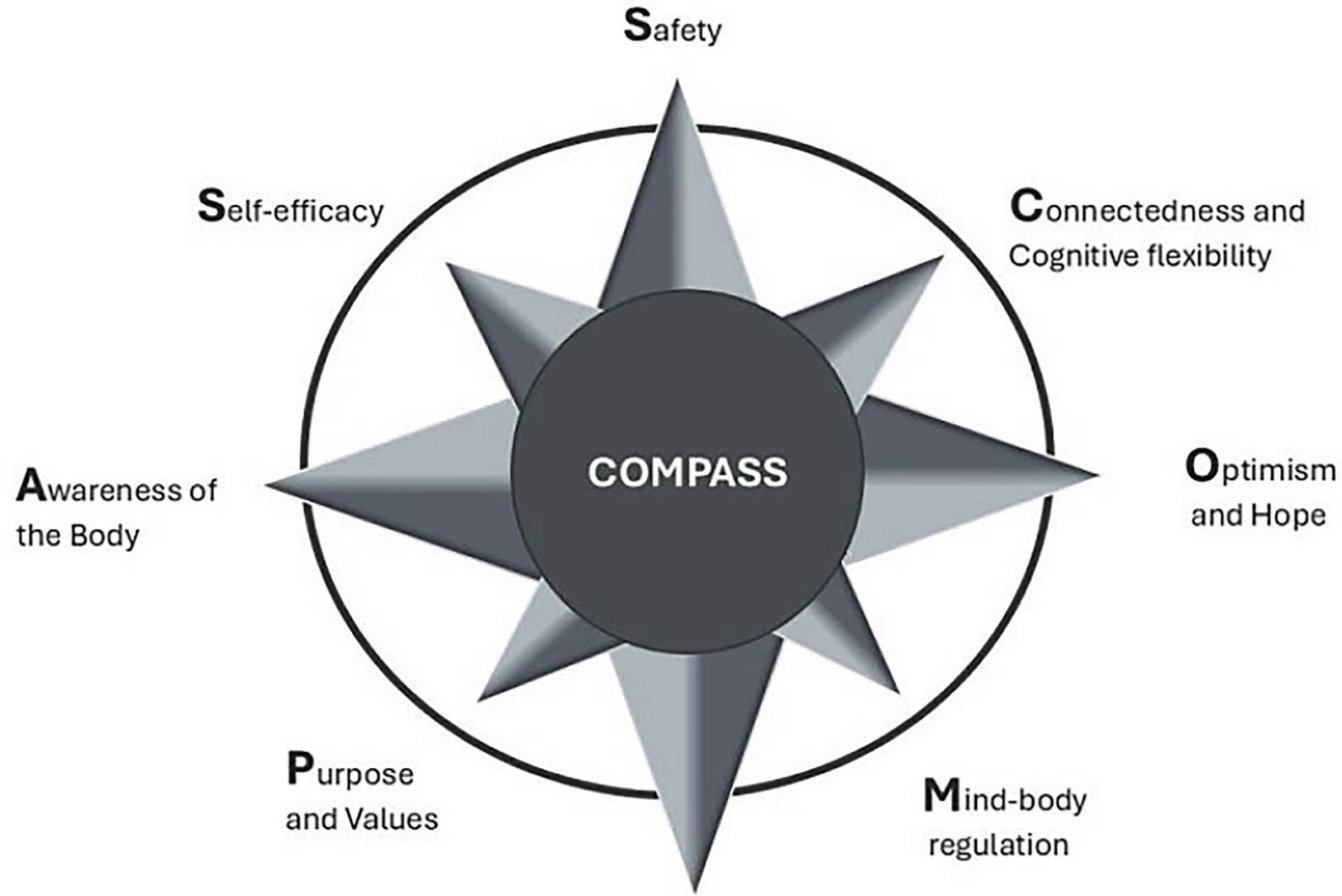
This figure illustrates part of the COMPASS framework, which identifies important factors supporting the cultivation of a sense of safety for individuals with chronic pain. It fosters processes of connectedness and cognitive flexibility, optimism and hope, mind–body regulation, purpose and values, awareness of the body, and self-efficacy, to ultimately support overall safety and increase resilience while living with chronic pain and PTSD.

Salutogenesis is a model of health promotion that embraces complexity and emphasizes the factors leading to whole-person well-being with a focus on factors contributing to flourishing and improved quality of life across dimensions of the whole person (28, 29). It is an alternative model to traditional pathogenic, disease-focused healthcare perspectives. Fostering a sense of safety is one route to whole-person well-being (30). Safety is multifactorial and has aspects germane to CP + PTSD. This study provides a framework (Figure 1, COMPASS) of yoga practice applied for CP + PTSD that supports one’s movement toward safety by catalyzing processes of Connectedness and cognitive flexibility, Optimism and hope, Mind–body regulation, Purpose and values, Awareness of the Body, and Self-efficacy and Safety. Complex populations such as those with CP + PTSD necessitate this sort of transdiagnostic and transdisciplinary approach in order to move beyond silos of physiological, psycho-emotional, social, and spiritual care. The sense of safety developed via the domains of the COMPASS framework will be presented in this article as a salutogenic framework for CP + PTSD.

### 1.1 Chronic pain and posttraumatic stress disorder phenotypes

Chronic pain is a condition defined by the presence of pain on more than half the days in the previous 3–6 months with substantial consequences in activities, functional disability, and/or significant emotional distress (31). Posttraumatic stress disorder (PTSD) is characterized by symptoms of intrusive re-experiencing of a traumatic event, avoidance of associated stimuli and reminders, alterations in mood and cognitions, and persistent physiological arousal and reactivity, which impact functioning and persist for at least one month following exposure to a traumatic event (5).

Chronic pain and PTSD each have variations in their clinical presentation. This has resulted in three distinct phenotypes for chronic pain: nociceptive, neuropathic, and nociplastic. Three to five distinct PTSD subtypes with neural correlates have been examined in pertinent literature (8–11). For PTSD, there is no single universally agreed-upon number of subtypes, but a host of literature has examined the observable differences and attempted to classify PTSD subtypes in different ways (e.g., dissociative features and neural biomarkers) (8, 32, 33). We find it important to acknowledge and briefly review these varying clinical presentations as they are germane to the presentation and application of our COMPASS framework.

The gate control theory and pain neuromatrix models describe pain as a product of the brain and/or of central and peripheral sensitization (34–36). The International Association for the Study of Pain (IASP) defines pain as “an unpleasant and emotional experience associated with, or resembling that associated with, actual or potential tissue damage” (37). By this definition, pain is not solely based on harmful or painful sensation (i.e., nociceptive stimuli) but is influenced by biopsychosocial factors and learned experience, a critical component of the clinical picture (37).

Three mechanistic phenotypes for pain are defined in the recent literature and by IASP: nociceptive, neuropathic, and nociplastic (38, 39). Nociceptive pain is caused and maintained by ongoing input from the tissue (e.g., mechanical, chemical, and thermal). Neuropathic pain derives from dysfunction within the nervous system. Nociplastic pain involves abnormal nociceptive and sensory processing and altered pain modulation without evident or clear cause of threat to tissues (38, 39). These phenotypes guide clinical decision-making and highlight three pathways contributing to the experience of pain (39). Nociplastic pain is of particular relevance to the discussion of CP + PTSD. The nociplastic phenotype includes a shift in pain signaling whereby the nervous system alters how it receives, integrates, and responds to stimuli (40–42). Safe or harmless stimuli begin to activate nociceptive and sensory pathways (e.g., “sensitization”), creating a mismatch between the stimulus and response, so that non-dangerous stimuli are perceived as threatening. Sensitization is one link in the relationship between psychological factors, such as experiencing trauma, and the physiological changes of altered pain signaling (10).

**Figure 2:**
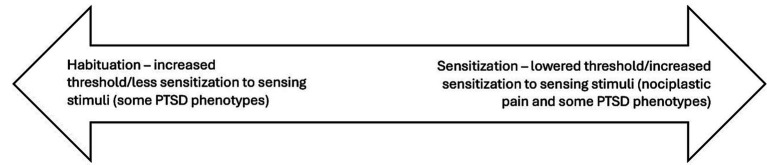
This figure depicts a continuum of sensation. The habituation end describes an increased threshold for sensation, resulting in less sensitization to stimuli. Some PTSD subtypes are prone to habituation. The sensitization end describes a lowered threshold for sensation, resulting in more sensitization to stimuli. Nociplastic pain and some PTSD phenotypes are prone to sensitization.

The activation of the sympathetic nervous system (SNS) interrelates with this altered pain signaling. As non-threatening stimuli are misperceived as dangerous, the nervous system may become weighted towards the perception of threat, resulting in SNS activation. Additionally, the dysregulation of the autonomic nervous system (ANS) towards SNS (activating) dominance, seen in people with chronic pain or PTSD, may prime the nervous system towards changes in sensitization (43, 44). Changes in nervous system signaling also occur with PTSD, where, over time, following exposure to trauma, shifts in neurobiology result in misperception of innocuous stimuli and corresponding threat perception responses (e.g., fight, flight, freeze) (45, 46). These changes in pain signaling are not simply in one direction (e.g., towards sensitization). Rather, they occur along a continuum illustrated in Figure 2, with one end representing an increased sensation threshold (habituation) and the other end representing a lowered sensation threshold (sensitization).

While the Fifth Edition of the Diagnostic and Statistical Manual of Mental Disorders (DSM-5) outlines emotional and behavioral PTSD symptom criteria, a body of literature examining its heterogeneity proves complexity well beyond those symptoms (8–10). The biological pathogenesis of PTSD implicates psychophysiological, endocrinological, genetic, molecular, and brain structural components with corresponding cognitive, emotional, and behavioral changes (45). Related, the 11th Revision of the International Classification of Diseases (ICD-11) contains an additional PTSD diagnosis with symptomatology characterized by disturbances in self-organization (i.e., affective dysregulation, negative self-concept, and disturbances in relationships) [i.e., Complex PTSD (CPTSD)] (47). The complexity surrounding PTSD has been borne out in the literature on CP + PTSD such that its diagnostic nuance shows evidence of different patterns in chronic pain symptoms and treatment responses (10, 12, 48). There may be either habituation or sensitization in physical or emotional pain threshold changes that can be determined by the mechanisms and/or levels of attention or dissociation related to the underlying trauma (e.g., PTSD subtypes) (48). For example, in combat-related PTSD or dissociative PTSD subtypes, there may be reduced conscious attention to stimuli and a bias towards habituation to sensation, or decreased pain sensitivity/increased pain threshold/less sensitization, respectively (48). On the other hand, in accident-related PTSD or traumatic events involving more anxiety, pain catastrophizing, and less dissociation, there is a bias in sensitization to the sensation end of the continuum (Figure 2), or increased pain sensitivity/decreased pain threshold/more sensitization (10, 12, 49). One study examined individuals with chronic pain and found higher rates of current pain and sensitization among those with PTSD, and also compared chronic pain subgroups based on sensitization levels and found those with higher levels of sensitization reported higher rates of childhood maltreatment and lifetime abuse severity [e.g., events that can produce dissociative symptoms (50)].

Given the intricate, complex nature of CP + PTSD, it is an important challenge to identify interventions that meet the person with CP + PTSD where they are on the habituation-sensitization continuum and tailor treatment accordingly. This might mean bringing awareness to sensation itself (i.e., addressing habituation) or reinterpreting non-threatening sensation as safe (i.e., addressing sensitization). Yoga practices can develop body awareness (i.e., interoception) to support pain signaling changes along this continuum (51, 52).

### 1.2 Frameworks and models for co-occurring chronic pain and PTSD (CP + PTSD)

The biopsychosocial model shifted the disease paradigm from the biomedical model towards a comprehensive framework that has become the most utilized theoretical perspective to date, with great utility for interdisciplinary care (17). Biopsychosocial assessments consider a holistic view of a person’s health through its biological, psychological, and social domains, arriving at a conceptualization of a person to inform treatment and recovery. It is necessary to include the biopsychosocial model in the immediate discussion due to its ubiquity; however, it is important to note that the biopsychosocial (and later biopsychosocialspiritual) model is inconsistently described as an approach, framework, model, and/or theory depending on its context and with implications in practice. Further, its critics argue that it is scientifically and theoretically empty (53, 54). Attempts to address this critique enhance the causal interactions between its domains and outline research paradigms (55).

Cognitive-behavioral therapy (CBT) is a broad approach to the conceptualization and treatment of mental and behavioral health that can be tailored to disorder-specific symptoms. The overarching theory of CBT is that restructuring maladaptive thinking patterns can lead to cognitive, affective, and functional restoration (56). Three prevailing cognitive-behavioral models of CP + PTSD, described below, share a focus on the overlapping and mutually reinforcing symptoms of these two conditions.

In the Mutual Maintenance (MM) model of CP + PTSD, shared underlying cognitive, emotional, behavioral, and physiological mechanisms cause an interplay of symptoms that mutually reinforce one another, maintaining the existence of both conditions (57, 58). Attention to physiological symptoms of anxiety or pain, thoughts that evidence worry or concern, or the misperception of harm, damage, or danger are biased stimuli pertinent to CP + PTSD, driving their mutual reinforcement.

The Shared Vulnerability (SV) model of CP + PTSD proposes that an underlying anxiety sensitivity predisposes one to respond with increased fear to physiologic sensation (58). Pain sensations and hyperarousal symptoms (e.g., increased heart rate or breathing related to PTSD or chronic pain) can produce anxiety responses. This is based on research that shows anxiety sensitivity is elevated in patients with PTSD and in some samples of patients with chronic pain (59, 60). The role of sensitization, where pain signaling becomes amplified in the central nervous system, has recently been proposed as an underlying mechanism for shared vulnerability (61–63).

The Fear Avoidance (FA) model identifies a cycle of catastrophic thinking leading to fear, hyperarousal, and avoidance of feared stimuli (64, 65). For chronic pain, fear and avoidance of movement limit daily life and social activity, resulting in a cycle of fear and negative affect/depression, increased disability, and diminished functioning (66). The relationship between fear and avoidance is well established for PTSD, where hypervigilant thinking, fear, and avoidance of trauma reminders result in the same cycle of consequences (64).

Cognitive-behavioral models of CP + PTSD have been augmented to acknowledge shared biological mechanisms (e.g., neurobiological and inflammation), co-developmental concepts and perspectives, meaning-making, and existential anxiety (12, 19, 20). In biobehavioral augmentations to existing models, brain processes, neurohormones, neurotransmitters, genetic polymorphisms, and inflammatory system factors are implicated (20). These processes include stress and fear conditioning, blocking extinction of conditioned stress, and impaired cognition and emotion regulation, all of which impede the recovery potential of therapeutic interventions (20, 67, 68). The Conceptual Model of Co-Developmental and Mutual Maintenance of Chronic Pain and PTSD highlights the influence of numerous biopsychosocial factors as they contribute to CP + PTSD development in pre-traumatic, peri-traumatic, and post-traumatic phases (12). The Integrated Model of CP + PTSD captures meaning-making processes (19). The identification of cognitive/affective, behavioral, and biological mechanisms of dysfunction informs existing evidence-based treatments like psychotherapy, medication, and mindfulness-based approaches.

### 1.3 Yoga: a salutogenic approach

**Figure 3:**
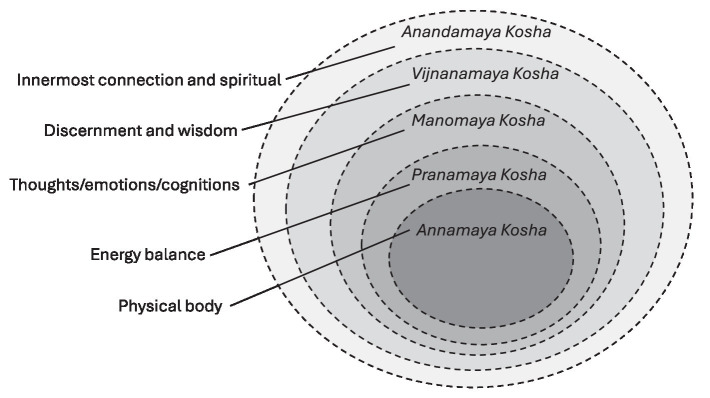
This figure illustrates the *Koshas* with a series of overlapping and interconnected circles, each representing a different aspect of one’s being in yogic philosophy: physical, energy levels and balance, thoughts/emotions, wisdom, steadfast contentment, or inner joy. The five *Koshas* provide a framework to understand the interconnectedness of the body, mind, and spirit. Yoga practices work with each layer to achieve balance and inward awareness.

Yoga is an ancient practice with origins in the region now known as India (69). While yoga is often thought of as a set of practices like breath regulation or movement, it is more accurately described as a philosophical path with specific practices that support an intention toward understanding contributors to and alleviators of suffering (70, 71). Yoga is inherently salutogenic in that it supports well-being in all aspects of life (72, 73). The teachings describe layers (*koshas*) of experience (Figure 3), including physical, energetic (breath and vitality), psycho-emotional, discernment or wisdom, and underlying authentic self or steadfast contentment, which can either detract from or support whole-person well-being (10, 74).

The practices of yoga span across the *koshas* (e.g., movement, breathwork, mental practice, and applied philosophy) and support restored balance within and between aspects of the self, facilitating an ongoing salutogenic process. Yoga practices also support connection to a deeper, authentic quality of the individual, underneath or separate from the fluctuations of the body, energy levels, and mind (75). As one experiences this connection, they find an inner stillness, quietude, or steadfast contentment, even in the context of ongoing states of chronic disease or dysregulation. Yoga practices support a person in developing a capacity to observe and discern their inner truth vis-à-vis the constantly changing body sensations, emotions, thoughts, and stimuli of the outer world. Through this discernment, yoga practices can be used to support regulation and resilience of the body, energy, and mind. In CP + PTSD, this connection to an inner sense of self, or stillness and quietude, provides a foundation for the experience of safety. This inner connection supports the whole-person system and offers opportunities to re-interpret non-threatening stimuli as safe. This may result in improved disease symptom severity, alongside increased acceptance of ongoing symptomatology.

Yoga practices include an 8-fold path of *yamas* and *niyamas* (ethical inquiry for valued living), *asana* (movement), *pranayama* (breath control and techniques), *pratyahara* (turning inwards as withdrawal of the senses from the outside world), and *dharana*, *dhyana*, and *samadhi* (representing stages of meditation from focused attention, sustained concentration, to absorption (71)). This rich practice includes both cognitive-based (e.g., top-down processing) and somatic-based (e.g., bottom-up processing) components (76). *Yama*, *niyama,* and meditation begin with a cognitive focus that has a downstream effect on physical well-being, whereas *asana* and *pranayama* start with somatic practice and have upstream effects for psycho-emotional well-being (77–80). The benefits of yoga through consistent practice of these limbs of yoga, including *asana, pranayama,* and various meditative practices (e.g., *mudra and mantra*), are essential to the system as a lived experience, rather than a more cognitively based philosophical pursuit (70). Yoga practices initiate the calming parasympathetic nervous system (PNS) in the *body first*, which reduces CP + PTSD hyperarousal of the SNS and aids in one’s ability to make changes to cognitive misperceptions of threat (e.g., bottom-up processing), which is critical for treatment (81). Yoga is a promising salutogenic intervention for CP + PTSD that includes mind- and body-focused practices (72, 73).

### 1.4 Summary of yoga and chronic pain

A growing body of literature demonstrates yoga’s effectiveness in managing chronic pain. Yoga is associated with improved pain, physical function, mental health outcomes, and/or quality of life in numerous pain conditions, including chronic low back pain, knee osteoarthritis, and rheumatoid arthritis (82–84). As such, yoga is now included in multiple clinical guidelines for the non-pharmacological and lifestyle management of different chronic pain conditions (85–87). Yoga interventions delivered in clinical trials for chronic pain populations tend to include gentle adaptive movement and poses, focused breathwork, mindful self-awareness, relaxation practices, and applied philosophy that facilitate cognitive reframing of disease symptoms and associated limitations. Mental health comorbidities such as depressive symptoms and psychological stress are common in chronic pain populations due to multiple mechanistic pathways (i.e., systemic inflammation, functional limitations, social isolation) and can create a reinforcing feedback loop by which mental health challenges exacerbate pain severity and interference (88). As a mind–body intervention with top-down and bottom-up practices, yoga may be uniquely poised to impact comorbid physical and mental health challenges associated with CP + PTSD (75).

When yoga is designed for a specific clinical population, as is done in clinical research trials, its safety is deemed equivalent to comparison groups, both active (e.g., exercise) and inactive (e.g., education), as demonstrated in a systematic review of randomized controlled trials (RCTs) with yoga as an intervention arm (89). Another systematic review of epidemiological studies, however, suggests that yoga delivered in community settings and not tailored to specific clinical concerns, such as yoga studios and recreational centers, may increase the risk of mild, transient musculoskeletal events (90). While most trials suggest improved chronic pain outcomes, some low-certainty evidence shows that yoga may increase the risk of pain exacerbation for some individuals (91). These findings highlight the need for clinical guidelines that address specific clinical risk factors, both physical and psychological, to optimize outcomes and avoid unintended harms.

### 1.5 Summary of yoga and post-traumatic stress disorder

In response to a growing demand for alternatives to psychotherapy and medication, yoga has been identified as a highly desired, beneficial intervention for the treatment of PTSD (92). Despite this, research on the efficacy of yoga for PTSD is inconsistent. Findings from individual RCTs show efficacy, which contradict findings from meta-analytic studies examining yoga as a therapeutic treatment for PTSD. These meta-analyses cite low-quality evidence for clinically relevant effects of yoga on PTSD compared to no treatment (25, 93). The flexibility of yoga limits systematic reviews and meta-analyses due to intervention heterogeneity, prompting the need for a broader look at the literature. A recent systematic review of RCTs that tested the efficacy of yoga for PTSD, assessed with a valid clinician-administered or self-report instrument, found that yoga participation improved self-report PTSD symptoms; however, the same was not found when change was measured using clinician-assessed symptoms (94). Further, the type of yoga differentially predicted outcomes, with those assigned to trauma-sensitive, *Kundalini*, *Satyananda* yoga, and a holistic yoga program faring better relative to a comparison group (94). Qualitative studies add context to the use of yoga as a treatment for PTSD. Participants from one interview-based qualitative study reported improvements in the mind–body relationship, emotion regulation via improved present moment awareness, cessation of negative thinking patterns, and taking action rather than avoiding stressful situations (95).

Down-regulation of nervous system activity is a theorized biological mechanism of action of yoga for PTSD (96). Untreated PTSD contributes to ANS dysregulation and, over time, contributes to cumulative stress, causing eventual dysfunction of metabolic and endocrine functioning and resulting in an increased risk of chronic diseases (21, 97, 98). Systematic reviews and meta-analyses of yoga show it counteracts the impact of cumulative stress by decreasing activity of the activating and arousing branch of the ANS (99, 100). Psychological mechanisms of action include increased choice and empowerment, distress tolerance/emotion regulation, embodiment and interoception, relaxation, awareness and acceptance of the present moment, and reduced avoidance (101, 102).

The terms “trauma-sensitive yoga” or “trauma-informed yoga” (TSY/TIY) refer to a gentle approach to yoga that considers the impact of PTSD symptoms. This style of yoga modifies the instruction, classroom, and yoga curriculum (e.g., omitting hands-on correction and using language of invitation) (103). A large-scale, multisite, comparative effectiveness RCT of a protocolized version of TSY versus a first-line psychotherapy for PTSD showed reductions in PTSD symptoms for both treatment groups from baseline to three months post-treatment, without significant differences between groups (93).

The body of empirical literature examining yoga for treating PTSD is limited by the heterogeneity of interventions, small sample sizes, and a lack of rigor in trials, with few trials comparing yoga to frontline treatments. Despite this, it has been recommended as an adjunctive or complementary intervention as part of multimodal or first-line therapies for the treatment of PTSD; dismantling studies are needed (104–106). Qualitative studies and systematic reviews show benefits, with systematic reviews also noting the limitations of the literature (94, 107, 108). Because of the multifaceted presentation of PTSD, a full scope of the literature on the impact of yoga for PTSD is warranted to better understand whole-person and functional improvements that are not captured by simply measuring PTSD symptom change.

### 1.6 Yoga for co-occurring chronic pain and post-traumatic stress disorder

Yoga is well-suited for the complexity of CP + PTSD given its inherently whole-person, salutogenic framework and its support for the alleviation of suffering through connecting to one’s essential nature, values, and purpose (70–73). These effects are best catalyzed when yoga is provided through its own framework, combining mind- (e.g., ethical principles of *yama/niyama* and meditation) and body-based (e.g., movement and breathing techniques) practices (75, 76, 109). Rather than focusing on one symptom or one mechanism, yoga has the potential to influence underlying factors that have concurrent effects on all aspects of well-being.

Physiological factors important to CP + PTSD and influenced by yoga practice include structural and functional neural changes, such as interoceptive skill-building, parasympathetic balance of the ANS, and improved endocrine and immune responses and inflammatory biomarkers (99). The support of ANS regulation is itself a salutogenic driver of whole-person well-being as it simultaneously shifts physiological systems and psycho-emotional states or qualities, all of which support social connection (110–113). Across clinical populations, yoga has been shown to support autonomic regulation, and the inclusion of ethical qualities (*yamas* and *niyamas*), such as non-harming, within the practice has shown increased effect for parasympathetic-driven autonomic and endocrine changes (75, 96, 109, 114, 115).

Interoception is the ability to sense the body’s internal state, which supports processing, interpreting, and regulating signals and sensations from within the body (116). As mentioned above, phenotypes and subtypes of chronic pain and PTSD include a continuum from habituation to sensitization for sensing internal and external stimuli. Supporting interoception is one way that yoga is thought to be supportive of rebuilding an adaptive, accepting mind–body connection for physical and mental health (117, 118). Yoga’s emphasis on awareness of mind–body sensation with non-judgment or non-reactivity can support new relationships with the body, as well as confidence with movement. Yoga practices can be used to support the spectrum of interoception, including noticing sensation, re-labeling sensation (i.e., describing something as “heavy” or “stinging” versus “pain”), re-establishing a relationship of thoughts and emotions to bodily sensations, reappraising safe sensation as non-threatening, methods to regulate the body–mind, and trusting the body. These practices can be applied with precision to support the reprocessing of sensation.

Yoga has also demonstrated effects in supporting social connection, a sense of purpose and meaning, and self-compassion, all of which support physical and mental health outcomes and are critical to CP + PTSD rehabilitation (119–122). Connection to purpose, meaning, and one’s values and relationships can influence how one experiences pain or trauma and is related to a myriad of physical and mental health benefits, including decreased all-cause mortality (72).

We know of only one pilot study examining yoga for reducing symptoms of CP + PTSD (123). Findings showed trend-level reductions in overall PTSD, as well as in symptom cluster scores for negative alterations of cognitions and mood, and arousal and reactivity (123). The study found significant improvement in fear of movement and ability to participate in social activities as pain-related outcomes (123).

## 2 The compass framework for chronic pain and PTSD

### 2.1 Yoga practices and their healing potential for co-occurring chronic pain and PTSD

**Figure 4:**
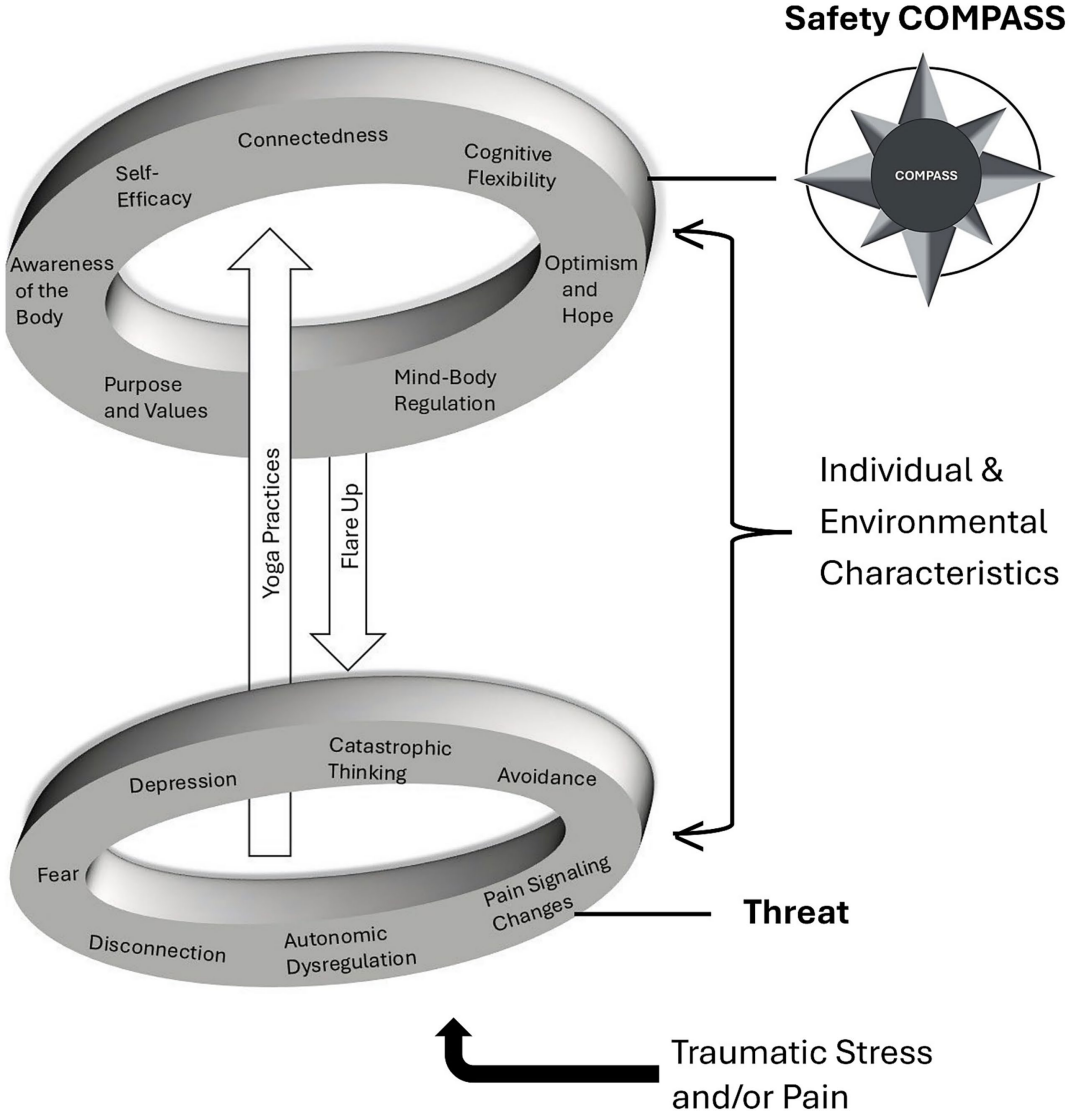
The COMPASS model for chronic pain and PTSD (CP + PTSD) and yoga represents a salutogenic picture, providing internal direction and navigation toward a whole-person experience of safety from threat. The framework in Figure 4 illustrates ways that a person with CP + PTSD may have their life experience weighted towards threat. This includes autonomic dysregulation, catastrophic thinking, fear, avoidance, depression and disability, pain signaling changes, and disconnection. Yoga, when provided within its cohesive and full framework, offers a system of practices to support a movement towards safety and foster resilience. COMPASS represents the tools that can be provided by yoga to support this navigation, including: connectedness, optimism and hope, mind–body regulation, purpose and values, awareness of the body, and self-efficacy, with safety being the cumulative result. While there may be flare-ups, influenced by a number of individual and environmental characteristics, which bring up transitory threat, the person is empowered in their resilience to return to safety.

The COMPASS Framework describes a process whereby traumatic stress and pain can increase a person’s experience of perceived threat. For individuals with CP + PTSD, the clinical picture can include any combination of autonomic dysregulation, catastrophic thinking, avoidance, depression, fear, and disconnection. Yoga practices help steer one towards safety and resilience by supporting Connectedness and cognitive flexibility, Optimism and hope, Mind–body regulation, Purpose and values, Awareness (body awareness and interoception), Self-efficacy, and Safety (COMPASS). Figure 4 illustrates the COMPASS framework and the dynamic nature of CP + PTSD, whereby a person may move back and forth between safety (upper circle) and threat (lower circle) due to flare-ups and recovery.

### 2.2 Threat

People with CP + PTSD experience life in a near-constant state of perceived threat that has biological (e.g., hyperarousal), psychological (e.g., catastrophic thinking or fear), behavioral (e.g., avoidance), and spiritual (e.g., disconnection) sequelae. These distort the nervous system’s response and have whole-person consequences, impacting identity, functioning, and quality of life (12).

From a state of threat, the body–mind seeks to prepare and protect itself in response to actual or perceived danger. The consequences of doing so comprise the lower circle titled “Threat”: autonomic dysregulation, catastrophic thinking, fear, avoidance, depression, pain signaling changes, and disconnection. These consequences are interconnected and occur to different degrees for each unique person living with CP + PTSD, relative to individual and environmental characteristics. They do not occur in a linear, sequential fashion.

#### 2.2.1 Autonomic dysregulation

The defense cascade model defines a continuum of responses to subconsciously perceived threat, including arousal, fight, flight, freeze, collapse, and immobility (124). The underlying autonomic neural ‘platform’ communicates with higher brain centers to create a quick, efficient physiological, psychoemotional, and behavioral response to threat. This dysregulation contributes to the sensitization of the nervous system and may mediate the association between some forms of trauma and pain severity (43, 44). Dr. Stephen Porges coined the term *neuroception* to describe how the ANS continuously surveils the environment to determine how the body responds along a relaxation-mobilization continuum (110).

The state of the ANS influences how we notice, evaluate, and respond to bodily stimuli (e.g., sensations), mental stimuli (e.g., thoughts and feelings), or environmental stimuli (e.g., sounds and smells (110, 111)). The ANS is heavily implicated in CP + PTSD, where the SNS, which broadly acts to arouse the body and mobilize for “fight-or-flight” action, becomes perpetually activated, driving the whole-person system towards threat and causing chronic imbalance with the PNS, which broadly acts to relax and soothe the body (43, 44, 125–129). Physiological, psychological, and behavioral changes occur in response to sympathetic activation from perceived threat (30, 110–112).

#### 2.2.2 Catastrophic thinking

Catastrophic thinking, or catastrophizing, is emblematic of the cognitive symptoms of CP + PTSD and refers to appraisals of threat and safety via stimuli interpretation. Catastrophizing can be ruminative, magnifying, and helpless in nature, creating worst-case-scenario thinking patterns (130, 131). Catastrophic thinking functions as a lynchpin in the maintenance of CP + PTSD due to its significant correlation with all symptom clusters of PTSD, negative beliefs about the self and identity, and fear of pain, pain intensity, and pain disability (132, 133). Pain catastrophizing has been shown to mediate the effect of PTSD symptoms on pain intensity. Similarly, PTSD symptoms mediate the relationship between pain severity and pain catastrophizing, indicating a synergistic effect of pain and PTSD, as captured in existing models of comorbidity (134). Pain catastrophizing has also been identified as a predictor of early treatment dropout (135). Fortunately, catastrophizing can be reduced following treatment (136).

#### 2.2.3 Fear

Fear of traumatic reminders or pain triggers dominates the cognitive and emotional profile of individuals with CP + PTSD and is a prominent component of comorbidity models (137). Fear has been identified as both a risk factor in the development of CP + PTSD and a factor in the maintenance of this comorbidity (12). Fear learning is rapid, persistent, and generalizes. Because it entails physiological arousal and behavioral consequences, it is difficult to reverse (138). Fear of pain is elevated in adults with trauma-related stress (139). Inhibiting fear responses is compromised in individuals with PTSD, and studies show that this impacts the ability to attenuate pain perception and treatment outcomes (140, 141). Safety learning may be enhanced by repetition, resourcing, and grading tasks to assist with learning processes.

#### 2.2.4 Avoidance

Intricately linked with fear and anxiety, avoidance behavior is a hallmark characteristic of CP + PTSD. Catastrophizing, fear-avoidance beliefs, and avoidance behavior form the psychological triad of CP + PTSD. When a person consistently encounters stimuli they perceive to be threatening or dangerous, a natural protective response is to avoid that stimulus (142). Avoidance works in some cases to prevent emotional or physical pain but generalizes over time. In doing so, the person with CP + PTSD often experiences a limited life, marked by isolation, negative mood, depression, and disability. Life becomes constricted and lacks vitality, connection with others, and positive emotional states (142, 143). Avoidance may spare an individual with CP + PTSD some encounters with hurtful stimuli, but it compromises opportunities to learn anew—that they can encounter such stimuli without it doing additional *harm*, and that the harmless hurt is something they can learn to tolerate and often reframe.

#### 2.2.5 Depression

Depressed thinking and mood are experienced by individuals living with CP + PTSD. Symptoms of depression include ruminative thinking and negative views of self, others, and the world. Depression can lead to isolation and disconnection, and when co-occurring with CP + PTSD, the consequences for the whole person are amplified. Over time, avoidance and isolation result in reduced functioning and disability (143). People with CP + PTSD experience more depression than those with either condition alone (144). Depression acts as a mediating variable between PTSD and pain symptoms, increases the misuse and abuse of substances, and interferes with the ability to divert attention from pain or to have a non-judgmental/accepting stance towards pain experiences (145–147).

#### 2.2.6 Pain signaling changes

The previous discussion on pain and PTSD phenotypes and subtypes highlights the spectrum of pain signaling changes that can occur in CP + PTSD. In nociplastic pain, safe stimuli can be misperceived as threatening as part of a process of sensitization of the nervous system (40–42). In sensitization, a decreased pain threshold magnifies innocuous sensations, eliciting a body–mind-behavioral response that amplifies threat. As a result of a nervous system activated towards threat, the person may exhibit fear-avoidance behaviors as they retreat from stimuli that activate the threat response (40, 42, 148, 149). This sensitization may contribute to or be affected by autonomic dysregulation as part of the clinical picture (43, 44). Individuals with CP + PTSD often demonstrate some level of sensitization, which can be either habituated or sensitized depending on specific mechanisms or levels of attention/dissociation associated with the individual’s PTSD experience and symptoms (150–152). Treatment for CP + PTSD should be informed by exploring the continuum of habituation and sensitization, which may differ when these conditions occur separately (48).

#### 2.2.7 Disconnection

The above-described whole-person experiences of individuals with CP + PTSD result in an overall, profound experience of disconnection from self, community/others, the world, values, and beliefs. This may be especially pronounced when symptom severity limits engagement in meaningful roles, such as caregiving, career-related activities, and service opportunities that involve connection with others (153, 154). Within-person disconnection may be experienced through a schism between body and mind or between separate aspects of either one (e.g., a site of chronic pain feels separate from the rest of the body) (155, 156). Related to spiritual matters, there may also be a sense of disconnection manifested as mistrust or feelings of abandonment by a previously trusted Higher Power (157, 158).

### 2.3 Safety

The whole-person, salutogenic recovery potential of regular engagement with yoga practices is described below and illustrated in the components of the “Safety” circle of the COMPASS framework. Relevant literature examining the sense of safety has described it as “transdisciplinary” and a “whole-person approach to care.” In CP + PTSD, where the whole is greater than the sum of its parts, it is paramount to engage a framework that represents a salutogenic approach to complex clinical populations, like those with CP + PTSD. In the COMPASS framework, Connectedness and Cognitive Flexibility, Optimism and Hope, Mind–body regulation, Purpose and Values, and Self-Efficacy are drivers shifting the system towards Safety.

#### 2.3.1 Connectedness

A sense of connectedness connotes integration of the whole body, a body–mind connection, an integration of the individual within the larger family and/or community structure, and potentially a feeling of spiritual connectedness with all humanity, nature, or a trusted Higher Power. In biomedicine and mental health fields, this can be conceptualized as a connection between aspects of the biopsychosocial-spiritual model (body, mind, family/community, spiritual life). In yoga, it can be understood as connection between the layers of the *Panchamaya Kosha* model (physical, energetic, mental/emotional, wisdom, spiritual Self). Whereas the lived experience of CP + PTSD can be associated with reduced connection and/or dissociation, body–mind practices such as yoga can work through top-down (meditation, mindfulness) and bottom-up (movement, relaxation practices) approaches to restore integration and connectedness.

#### 2.3.2 Cognitive flexibility

The ability to shift one’s attention, adapt to new information or changing situations, and change one’s mind accordingly is known as cognitive or psychological flexibility. Cognitive flexibility is one of the primary facets of executive functioning, the brain’s ability to engage higher reasoning and problem-solving skills. Rigid thinking patterns, such as catastrophic thinking, are mental shortcuts emblematic of CP + PTSD. Increasing cognitive flexibility is the pinnacle of cognitive control and is intrinsically linked to adaptive behavior, which is critical for overriding habits of thinking, feeling, and behaving to make whole-person changes to functioning and well-being (159). Restructuring thinking from rigid to flexible is the cornerstone of modern psychotherapy, and successfully doing so can produce improvements in affect, behavior, and functioning (160). Specifically, improving cognitive flexibility has been shown to predict improvements in self-efficacy, daily functioning, self-compassion, and symptom improvement for CP + PTSD (161, 162). Yoga’s ability to engage the PNS can positively influence stress hormones known to diminish cognitive flexibility. Specifically, there is strong empirical support linking the practice of yoga *asana* (i.e., poses/postures) and *pranayama* (i.e., yoga breathing practices) with attributes that support cognitive flexibility (163–165). Yoga has been shown to improve psychological flexibility, making it a powerful tool for cultivating Safety (166).

#### 2.3.3 Optimism and hope

These related constructs indicate an orientation toward what is possible, rather than what is limited. Hope suggests a belief that something could change for the better. In CP + PTSD, this may be an improvement in symptoms or a reduction in the impact of symptoms on function, roles, and quality of life. It serves both as a noun and a verb in that hope can be an active, goal-oriented process. Optimism reflects a general positive outlook oriented towards what can go well, rather than potential negative outcomes. In psychotherapy, tools such as cognitive reframing support a shift toward hope and optimism by changing personal narratives and perspectives (167, 168). In yoga, the practice of *pratipaksha bhavana,* or “cultivating the opposite,” focuses on discontinuing negative ways of thinking and replacing negative thoughts with more positive ones (169). This is not to ignore real challenges associated with CP + PTSD, but to recognize that even in the context of challenging conditions, positive experiences are possible and can be fostered intentionally.

#### 2.3.4 Mind–body regulation

Supporting mind–body regulation is key to perceiving and experiencing safety (30, 110, 112). Methods like vagal nerve stimulation, biofeedback, and yoga support a parasympathetic dominant system (i.e., the capacity to rest and digest) and are critical for mind–body regulation in people with CP + PTSD (170–172). Yoga combines mind and body practices within its system, thereby providing an integrated approach to whole-person regulation (32, 75, 76, 173, 174). These practices can be thought of as a ‘neural exercise’ to widen one’s window of tolerance for physiological, emotional, and environmental stimuli (175).

Dr. Porges’ Polyvagal Theory describes how the autonomic neural platform mobilizes the body, mind, and behavior in a unified manner towards safety (110, 111). When physiological safety is supported, a person has a greater capacity to reinterpret what is being perceived by the body and mind and to more accurately discern what is safe and what is dangerous (30, 176). This embodies salutogenesis and uncovers whole-person well-being by focusing on factors that contribute to flourishing and improved quality of life across all dimensions of the whole person (e.g., physiological, cognitive, emotional, social, and spiritual (28, 29)).

#### 2.3.5 Purpose and values

A salient experience of CP + PTSD is a diminished sense of meaning, purpose, or connection to what is of personal importance (19). There may be an existential shift as one learns to live with and manage their symptoms and condition. This sense of meaning can influence how one appraises their body–mind responses, including catastrophic thinking, avoidance, or fear; how one is able to regulate and work with pain signaling changes or autonomic dysregulation; and how one experiences disconnection from others or their sense of self and purpose in the world. Connection to values and purpose influences the sense of safety. The person may find a way to access greater body–mind regulation and reorientation to oneself to improve self-efficacy and an internal locus of control to manage symptoms and reconnect to life. The person may experience a sense of hope or optimism to connect to new ways of living authentically.

Connecting to one’s chosen values and supporting a purpose-filled life is at the core of yoga practices and is especially apparent in the practice of *yamas* and *niyamas* (72). The *yamas* and *niyamas* have been shown to increase physical and mental well-being and have been identified as key to incorporating the holistic system of yoga into practice (115, 177, 178). Yogic texts describe how these ethical principles are foundational to the practice of yoga and the aim to alleviate suffering (71, 179, 180). The context in which all yoga practices are provided is within the greater philosophical context of *dharma*—an alignment with meaning and purpose supportive of the individual and their relationship with others and the environment (72, 180). The inclusion of these components of yoga has shown increased effect for whole-person well-being, and motivators for continued practice have been seen to shift from the physical to these aspects that include values and meaning over time (e.g., social and spiritual components) (115, 120, 181–183).

#### 2.3.6 Awareness of the body

Distorted or disturbed body awareness often occurs in individuals living with CP + PTSD (33, 184–187). Interoception encompasses multifaceted aspects of body awareness, such as the ability to attend to one’s internal bodily state, notice the relationship of emotions to the body, and the capacity for system regulation (116, 118, 188). The Multidimensional Assessment of Interoceptive Awareness measures interoceptive skills and body awareness, including noticing, not worrying, regulating attention, emotional awareness of the connection between emotion and the body, self-regulation, body listening, and body trusting (189, 190). Yoga has been found to support body awareness and interoceptive skills, including aspects of noticing, attention, self-regulation, and body listening (51, 117, 118, 184, 191–193). The integration of cognitive-based regulation skills, such as attentional control, alongside autonomic and body-based regulation skills—through breath practices, body relaxation training, and purposeful and supportive cues for movement—supports these various facets of interoceptive skill building (75, 76). In chronic pain, diminished interoceptive skills have been associated with increased worry about bodily sensation and lower body trust. Movement-based contemplative practices are supportive of regaining sensory-motor integration, reconnection with the body, and body confidence (186, 194). Interoceptive accuracy weakens as part of living with PTSD, and new learning for exploring safety in bodily sensation is key to regaining confidence in the body by changing how one responds to and attends to internal sensations (81, 195).

Individuals with CP + PTSD may demonstrate an inability to feel sensation (habituation), requiring more skills of noticing, or they may exhibit physiological or emotional overwhelm in response to sensation (sensitization; Figure 2). Yoga practices address this challenge by increasing awareness of body–mind sensation for habituation through movement, breathing, and noticing relationships between emotions, thoughts, and bodily sensations. Additionally, for sensitization, yoga practices can support non-judgmental and compassionate noticing, investigating relationships between emotions and bodily sensations, reappraisal of sensation from threat to safety, and a regained trust in how to listen and relate to body sensations.

#### 2.3.7 Self-efficacy

Self-efficacy, or the empowering belief in one’s competence and ability to have agency in one’s life, improves as a person uses yoga practices to manage their CP + PTSD (196). As someone gains mastery over their emotions and their interpretations of events and memories, improves their ability to move their body, and trusts the messages it communicates by developing interoception, self-efficacy improves. A cascading effect where self-efficacy improvements reduce catastrophizing is also seen with regular yoga practice (197). Positive self-appraisal and positive affect can drive motivation and behavior in all domains of life and shift one from catastrophic, negative thinking patterns associated with being in the Threat cycle of CP + PTSD, to empowering ways of exploring body–mind-environmental experiences in the Safety cycle accessed by yoga practices.

Yoga practice often utilizes the language of exploration and invitation to assist with the shift from disempowerment to empowerment via choice-making (198). For example, asking the question, “What might it be like to bend your knees more in this position?” offers a yoga student the opportunity to choose to do so (or not) and gather more information about what happens if they do (or do not) bend their knees further. Over time, the repetition of making such choices has an empowering effect, cultivating self-efficacy and an internal locus of control (198).

### 2.4 Individual and environmental characteristics

The Wilson-Cleary Model, first proposed in 1995, suggests a link between underlying biological function and overall quality of life, mediated by biopsychosocial factors including disease symptoms, functional status, and general health perceptions (199). Each of these mediators is moderated by characteristics of the individual and characteristics of the environment. Based on this model, underlying disease states could result in vastly different quality of life, depending on the biopsychosocial mediators and moderators at play. For example, degenerative disc disease symptom severity varies widely and can inform the patient’s functional status regardless of disease severity (200–202). This then impacts general health perceptions, which can also be influenced by health beliefs and self-efficacy (203, 204). Depending on factors such as social support and sense of purpose, quality of life may be more or less influenced by those health perceptions. A systematic review of twenty-six studies that used or tested the Wilson-Cleary model supported linkages between its components, with symptom severity serving as the strongest predictor of quality of life (205). The authors concluded that the factors of the Wilson-Cleary model were suitable for inclusion in quality of life research conducted on chronic diseases.

In persons with comorbid CP + PTSD, there is a complex interplay between the two conditions, each of which is also informed by individual and environmental characteristics. In the above-cited literature, it is noted that degenerative disc disease is present in some asymptomatic patients. Conversely, chronic low back pain is often non-specific, meaning that there is no underlying tissue pathology to explain the pain symptomatology from a musculoskeletal perspective alone (206). The same low correlation can be found in PTSD, where severe trauma history may be associated with low symptoms, whereas mild trauma history can be associated with unbearable symptoms (207). Individual characteristics, such as personal risk tolerance, along with environmental characteristics, such as family culture, can modulate symptoms upward or downward. Complexity is added in that chronic pain can serve as an individual characteristic that influences PTSD symptoms, and comorbid PTSD can worsen pain.

### 2.5 Yoga practices and flare ups

CP + PTSD symptoms can fluctuate dramatically, sometimes in distinct episodes (i.e., a “flare-up” or “relapse”), resulting in sudden changes in functional abilities and negatively impacting daily activities (208). Yoga practices can be adapted for greater accessibility amidst changing physical and psychological needs. In instances of reduced physical function, yoga postures can be executed with props or modifications that reduce stress on the body and mitigate the exacerbation of fear, avoidance, and other CP + PTSD symptoms. Protocols for yoga practice that consider functional limitations characteristic of pain or PTSD can be offered for this population to assist with moving from the Threat cycle to the Safety cycle during such flare-ups (103, 209–211). Movements can be executed more slowly, and restorative practices can be selected to facilitate greater ease and comfort. This requires yoga providers with specialized training to identify participant needs and offer appropriate adaptations and support. During PTSD flares, greater consideration of trauma-informed practices may be incorporated to decrease dysregulation while promoting psychological safety (198). Such practices may include less-vulnerable pose variations, shorter periods of silence, emphasis on choice-based practice, and intentional room orientation, which may be constrained by setting (103, 198). While people with CP + PTSD may choose more physically or psychologically challenging practices outside of a flare-up, the availability of these options allows for sustained practice that meets the person where they are with accessible, relevant tools that can be adapted as needed. It is important to note that while pain can be a helpful indicator for self-selecting practice variations in yoga, there is only a weak correlation between pain severity and tissue damage for structural chronic pain conditions such as osteoarthritis (212). In such cases, pain signaling is one of several factors that should be considered, alongside general and personalized medical guidance. Additionally, though mindful self-awareness can foster greater discernment in modulating practice choices, it also carries some risk of exacerbating hypervigilance for those with CP + PTSD (213).

## 3 Discussion

In this study, we addressed the shortcomings of existing models of comorbid CP + PTSD and presented the COMPASS framework for cultivating whole-person resilience. The shortcomings of existing models have implications for treatment outcomes and leave many with CP + PTSD without a model for whole-person recovery. We reviewed the phenotypes and subtypes of chronic pain and PTSD and described the whole-person consequences and co-prevalence of CP + PTSD. Based on prior work examining CP + PTSD theoretically, conceptually, and clinically, existing comorbidity models and their limitations were reviewed. The case for yoga as a salutogenic approach was made, and research testing yoga for chronic pain and PTSD separately and together was summarized. This background justifies the gap within which we present the COMPASS framework, comprised of components illustrating the whole-person sequelae of living with CP + PTSD, labeled in the “Threat” circle, as well as whole-person salutogenic benefits achievable through yoga practices, labeled in the “Safety” circle. We synthesized the contribution of the Wilson-Cleary model into our framework by acknowledging individual and environmental influences on the dynamic experience of living with CP + PTSD. Finally, we discussed applications of the COMPASS model and yoga practices vis-à-vis the episodic, flare-up nature of CP + PTSD. Ultimately, a greater window of tolerance to experience symptoms of CP + PTSD and factors that may prime the whole-person system towards Threat can be accessed from this foundation of Safety.

### 3.1 Treatment implications

As a treatment for CP + PTSD, yoga can be applied as a standalone intervention or integrated as part of a multimodal, interdisciplinary, or multidisciplinary treatment. Individuals trained in yoga as a therapeutic intervention can be multidisciplinary health care providers, including yoga teachers, yoga therapists, occupational and physical therapists, or mental health providers cross-trained in yoga practices. Each of these providers offers unique perspectives, and their respective disciplines and training have implications for treatment.

#### 3.1.1 Treatment models and approaches

Currently, clinical guidelines for CP + PTSD do not exist. However, given the high co-prevalence of these conditions, the clinical practice guidelines for each condition emphasize concurrent/parallel, sequential, or integrated treatment when they co-occur, emphasizing psychotherapy and primary care interventions (15, 214–216). The state of the science of yoga for chronic pain supports its use as a front-line treatment; however, for PTSD, yoga is recommended as an adjunct to other gold standard treatments (85–87, 104, 105, 217). Notably, one recent well-powered study showed yoga for PTSD to be non-inferior to a gold standard trauma-focused psychotherapy (93). Only one study has examined yoga for this particular comorbidity and had promising results, though more rigorous research is warranted (123).

Interdisciplinary and integrated treatment emphasizing salutogenesis represents an aspirational goal for attaining whole-person recovery from CP + PTSD. Current treatment paradigms for CP + PTSD are pathogenic, and care is siloed by discipline or clinic, even in integrated care settings (216). For example, a qualitative study on CP + PTSD in the Veterans Affairs healthcare system identified that Veterans with CP + PTSD might be assessed by a primary care provider, referred to a pain clinic to treat chronic pain, and then concurrently or sequentially referred to a mental health clinic for PTSD treatment, creating a paradox of interference (216). In this paradox, symptoms of the untreated condition interfere with recovery from the prioritized condition. Siloed care risks burdening a patient with multiple appointments or causing confusion, as the treatment model implicitly communicates that these conditions are separate, which can be invalidating and frustrating. When a person with CP + PTSD receives concurrent care, the conceptualization and treatment plan of two different providers may conflict or create a burden for the person who is experiencing the whole-person impact of CP + PTSD but for whom care is compartmentalized, confusing, and burdensome (216). In multidisciplinary care, multiple providers from differing disciplines or specializations treat the same constellation of whole-person CP + PTSD symptoms from a unified conceptualization. For example, a person with CP + PTSD might see a sleep specialist, a pain specialist, a mental health provider for trauma, and a separate therapist for their neuropsychological symptoms (e.g., attention or memory complaints). Multimodal interventions are often multidisciplinary—for example, a person with CP + PTSD receives pharmacological intervention (e.g., analgesic medication) from a primary care provider and psychotherapy from a mental health provider to manage catastrophic thoughts and avoidance. In interdisciplinary and integrated care, the treatment team meets to discuss a unified conceptualization and approach to the care of the whole person, including consequences experienced by the patient with CP + PTSD. A more advanced treatment approach would integrate care, treating both conditions simultaneously, in a transdiagnostic and transdisciplinary manner, as yoga has the potential to do, illustrated by the COMPASS framework. It is important to note that this sort of change in the treatment paradigm must necessarily consider the system within which care is provided and the types of care and providers who are available.

#### 3.1.2 Yoga teachers and yoga therapists

Those with advanced training and experience in therapeutic yoga and yoga therapy are uniquely situated to work with individuals with CP + PTSD. They can fully apply a yoga methodology and framework to care, guided by the COMPASS framework. The yoga professional can begin through any of the practices of yoga (e.g., *yama* and *niyama, asana,* and *pranayama*). Other yoga practices can be introduced to support access to greater inner awareness, regulation, rest, or sleep. All of these practices can be used alone or in combination. Each practice offers an opportunity for the individual to gain self-efficacy and greater connectedness to their own body–mind system, and to experience greater connection with themselves and the world around them. Yoga practices can be taught by therapeutic yoga teachers in group classes or private sessions, adapted for accessibility and safety. Most yoga classes are not intended specifically for CP + PTSD populations and may trigger physical or mental health symptoms. Accordingly, referring providers or patients seeking yoga instruction should identify teachers and classes that are trauma and pain-science-informed. Yoga therapists, however, are specifically trained to conduct an individual assessment and develop a tailored care plan of yoga practices unique to the individual’s concerns, goals, and limitations. This approach considers physical and psychological safety and identifies practices best suited to restore balance in areas of greatest priority. A yoga therapist will also conduct periodic assessments of progress on clinical indicators and adapt the care plan accordingly.

#### 3.1.3 Physical and occupational therapy (PT and OT)

COMPASS offers guidance to PTs and OTs working with individuals with CP + PTSD. In physical therapy, the practice of psychologically informed care is growing, as is the emphasis on whole-person care to address the complex clinical picture of people with co-occurring mental and physical health concerns (218). Pain education is one part of pain care utilized by PTs and OTs; it involves the cognitive process of learning how pain changes the nervous system to perceive safe stimuli as a threat (41, 42, 219). Pain education aligns well with the COMPASS framework as it aims to reconceptualize sensation by teaching individuals ways to re-evaluate stimuli (40, 41, 219). In those with CP + PTSD, pain education has been shown to increase pain self-efficacy (i.e., supportive beliefs about pain, such as confidence that exercise is safe) and decrease PTSD symptomology (220). Limitations in this intervention include the reliance on more passive or didactic strategies versus active body-based learning techniques (219, 221–224). The COMPASS framework illustrates how pain education, as an embodied and experiential approach to build interoceptive skills, can assist in reconceptualizing sensation and cultivating safety, with additional improvements in self-efficacy and confidence for movement and body awareness. PTs and OTs, trained in therapeutic yoga or working with yoga teachers/therapists, can support this embodied pain education approach.

#### 3.1.4 Psychotherapy

Currently, psychotherapeutic approaches are front-line treatments for CP + PTSD, though this is a paradigm in transition, as many individuals with chronic pain continue to receive treatment from primary care providers. Further, those with chronic pain who are referred to mental health providers often perceive that this means the pain is “all in their head,” which can interfere with treatment engagement. A systematic review and meta-analysis of psychological interventions for CP + PTSD included 18 trials (7 uncontrolled and 11 RCTs) of exposure-, cognitive-behavioral, and mindfulness-based psychological approaches. Data from ten RCTs were available for meta-analysis and showed a moderate effect on reducing PTSD severity, and a non-significant effect for pain outcomes (intensity and interference (225)).

Given the efficacy of yoga for chronic pain conditions and psychotherapy for PTSD, we suggest that the next step in research is to examine somatically-informed psychotherapy, or the integration of interoceptive skill building alongside cognitive intervention (226). This might involve adding therapeutic yoga practices to psychotherapy provision or drawing on yoga practices such as *pratipaksha bhavana,* or “cultivating the opposite,” which assists people with CP + PTSD in changing catastrophic and negative thinking patterns. Ethical considerations of practicing within one’s scope of practice drive consideration of cross-certification in both therapy and yoga facilitation.

A small but developing body of literature examining integrated psychotherapy and yoga for comorbid conditions shows promise in combining the top-down benefits of psychotherapy with the bottom-up benefits of yoga (226–230). Specifically, the focus on interoceptive awareness is of critical importance for this particular comorbidity, and clinical frameworks emphasizing the development of interoception have been discussed (226).

### 3.2 Cultural considerations and special populations

Patient-centered care places the patient at the center of their treatment, as a partner in their own care, drawing on their preferences, values, and culture to guide medical decision-making (231).

While the prevalence of CP + PTSD is high in the general population, it is disproportionately high among Veteran populations (232). Because of this and the numerous deleterious impacts CP + PTSD has on Veterans, including a severely increased risk of suicide, much of the existing research on CP + PTSD has been conducted with Veteran populations (233). Recently, research on CP + PTSD in Veteran populations shows that non-pharmacological, non-psychotherapeutic, mindfulness-based interventions like yoga are highly desired and can act as a gateway, with a Veteran three times more likely to follow yoga participation with an evidence-based psychotherapy (92, 234).

Special considerations are appropriate for CP + PTSD across the lifespan. This includes both pediatric and geriatric patient groups. In pediatric populations, care must be taken to design and deliver developmentally appropriate intervention formats and delivery that includes caregivers as critical stakeholders who are impacted by the patient’s symptoms and healthcare experience (235). In geriatrics, multimorbidity beyond CP + PTSD is common, and patients may be impacted by additional mobility and/or cognitive limitations (236).

Chronic pain conditions vary widely, and those with an autoimmune component, such as rheumatoid arthritis and systemic lupus erythematosus, may be especially sensitive to stress-related flares, environmental exposures, and additional symptoms such as debilitating fatigue (237–240). Patients with chronic pain syndromes that are often misdiagnosed or dismissed, such as fibromyalgia, may have experienced prior healthcare-related trauma and may be particularly sensitive to messaging about the disconnect between tissue damage and symptom severity. While comorbid CP + PTSD is common in these populations, it is important to avoid assumptions about individual health history and receptivity toward whole-person healthcare approaches.

Because yoga is a philosophical and spiritual path that shares cultural context with Hinduism and Buddhism, it is frequently misunderstood as being part of one of these religious traditions, creating a barrier for some patients with CP + PTSD who may benefit from yoga’s therapeutic effects (241). Yoga providers can help to dispel such concerns and to increase accessibility with culturally concordant instruction and patient education about yoga history.

### 3.3 Limitations

This framework is not without limitations. CP + PTSD itself is a complex comorbidity. Our review of pain descriptors and PTSD subtypes, and CPTSD, highlights an additional layer of complexity that research has shown to have implications and warrants continued empirical examination relative to this framework (12). This first presentation of the COMPASS framework considers the current state of scientific knowledge and those aspects of chronic pain or PTSD that have already been examined in theoretical, observational, and experimental science. As this framework is applied in clinical and research settings, we anticipate its evolution, particularly as a result of the implications of previously reviewed pain descriptors and PTSD phenotypes and cultural considerations.

### 3.4 Future directions

The COMPASS framework for CP + PTSD draws on existing biopsychosocial, cognitive-behavioral, neurophysiological, and yoga models, acknowledging the nuances of both chronic pain and PTSD and addressing the gaps and limitations of existing models. The COMPASS framework is conceptual and, as such, can guide clinical practice and research. Next steps that apply the COMPASS framework are described below.

Clinically, when a provider encounters a treatment-seeking individual with CP + PTSD, this framework can be used to collaboratively assess their whole-person experiences. Even without using yoga to assist the person in moving from threat to safety, the Threat and Safety components of the model and explanation of individual and environmental characteristics can lead to an impactful realization, increasing the individual’s self-awareness and insight. This provider-patient moment has the potential to build rapport, as the provider bears witness to a whole-person experience that such an individual may not have previously encountered. The power of bearing witness cannot be understated as an intervention, especially for those with CP + PTSD who are prone to disconnection (242). For those unfamiliar with yoga practices, COMPASS can be an educational tool about yoga and its potential to cultivate resilience. This can be particularly beneficial for populations who may feel that biomedical and psychotherapeutic treatments they attempted or considered did not work or were not a good fit. This framework can serve as a compass and a map guiding care as the provider and patient communicate about the challenges and achievements involved in CP + PTSD treatment. It can be used to reflect on desired or actual changes. Finally, this framework can support a clinical reasoning process for integrating all yoga practices in context with their philosophical foundation. The provider can identify priorities within the threat circle and explore the practices and philosophies of yoga that can be utilized to support the movement to safety in the upper circle.

The COMPASS framework can also be used to guide research focused on CP + PTSD and yoga as a therapeutic intervention. It may also be applicable to other related mind–body practices (e.g., tai chi and qi gong). A next step is to employ qualitative research designs to foster exploration of the model prior to experimental testing. A Delphi study that engages subject matter experts—those with expertise in chronic pain, PTSD, and yoga—would provide a consensus on the applicability of this model as well as where it may need refinement. Quantitative research designs might then employ measures assessing the components of the Threat and Safety circles or assess individual and environmental characteristics via measures of health-related quality of life prior to and following completion of a yoga intervention. Mixed methods research designs, which combine qualitative and quantitative methodologies to better understand experiences that can be captured via validated and standardized assessment measures, versus those best understood via qualitative methods, are another promising avenue for future directions of the COMPASS framework.

## 4 Conclusion

In this study, we introduced the COMPASS framework for CP + PTSD and yoga by first addressing limitations and gaps in existing models, then providing justification for the integration of yoga into the clinical conversation around CP + PTSD, and finally, by describing the framework components in detail. This model draws on and synthesizes existing models, combining them to increase conceptual understanding of the whole-person lived experience of CP + PTSD and the salutogenic potential of yoga. It is our most sincere hope that this framework will benefit those living with CP + PTSD, those treating CP + PTSD, and those passionately dedicated to empirically understanding CP + PTSD to advance the science and treatment of CP + PTSD.

## Data Availability

The original contributions presented in the study are included in the article/supplementary material, further inquiries can be directed to the corresponding author/s.
